# Utility of a multigene testing for preoperative evaluation of indeterminate thyroid nodules: A prospective blinded single center study in China

**DOI:** 10.1002/cam4.3450

**Published:** 2020-09-25

**Authors:** Yuntao Song, Guohui Xu, Tonghui Ma, Yanli Zhu, Hao Yu, Wenbin Yu, Wei Wei, Tianxiao Wang, Bin Zhang

**Affiliations:** ^1^ Key Laboratory of Carcinogenesis and Translational Research (Ministry of Education/Beijing) Department of Head and Neck Surgery Peking University Cancer Hospital and Institute Beijing China; ^2^ Genetron Health (Beijing) Co. Ltd Beijing China; ^3^ Key Laboratory of Carcinogenesis and Translational Research (Ministry of Education/Beijing) Department of Pathology, Peking University Cancer Hospital and Institute Beijing China

**Keywords:** indeterminate cytology, molecular diagnosis, next‐generation sequencing, thyroid cancer, thyroid nodules

## Abstract

**Background:**

Thyroid nodules are highly prevalent, with fine‐needle aspiration (FNA) commonly used as the standard preoperative tool for their diagnosis. However, the method classifies some of the samples as indeterminate, leading to unnecessary surgery. In this study, we evaluated the value of next‐generation sequencing (NGS) for cancer diagnosis in indeterminate thyroid nodules.

**Materials and methods:**

We performed a prospective, blinded cohort study on 189 patients, with 196 Bethesda III/IV nodules. Specifically, we analyzed DNA mutations and RNA fusions across the FNA samples using NGS, then reviewed follow‐up reports from 84 nodules following definitive surgery, to determine the assay performance.

**Results:**

Enough DNA and RNA were obtained in 188 nodules, revealing mutations or fusions in 34.6% of them. The most frequently mutated genes were RAS, followed by BRAF V600E. Based on surgical pathology, 39% (33/84) and 4.8% (4/84) of the nodules were malignant and intermediate, respectively. According to the risk stratification criteria, 28 cases were categorized High‐Risk group, all of the resected nodules (n = 20) were malignant. Twenty‐four thyroid nodules were in the Low‐Risk group, 28.6% (4/14) surgically removed nodules were malignant. In the Benign‐Like category, 18.0% (9/50) were malignant. Five out of 13 nodules with benign mutations were resected, including SPOP, EZH1, and ZNF148, all of them were benign. If genetic alterations annotated with High‐Risk or Low‐Risk was considered as positive, and negative if Benign‐Like. Multigene testing revealed sensitivity, specificity, positive predictive values (PPV), and negative predictive value (NPV) of 73%, 80%, 71%, and 82%, respectively. In addition, if four intermediate nodules were counted as malignant, the PPV and NPV were 71% and 74%.

**Conclusion:**

Our results allow for further stratification of Bethesda III/IV thyroid nodules based on the risk of their malignancy. SPOP, EZH1, and ZNF148 mutations may be used as benign markers.

## Graphical abstract

We introduced a multigene panal for cancer diagnosis in FNA‐indeterminate thyroid nodules, and evaluated its performance in Chinese population.

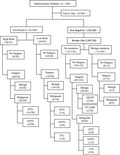

## 1 INTRODUCTION

Popularization of ultrasound examination has increased the prevalence of thyroid nodules in China and around the world. Generally, ultrasound‐guided fine‐needle aspiration (FNA) is the first line clinical test for detecting cancer in thyroid nodules, owing to the fact that a vast majority of thyroid nodules are benign.^1^ In a subset of cases, current cytological techniques fail to clearly differentiate between benign and malignant disorders.^2^ Previous studies have demonstrated that findings from about 10%‐30% of FNAs reveal an indeterminate state, necessitating surgical excision to rule out malignancy.^3^ In fact, the proportion of indeterminate cytological results appears to be rising.^4^ However, 70%‐80% of these indeterminate nodules are eventually confirmed to be benign lesions, meaning that surgery could have been avoided.^5^

In recent years, remarkable progress has been made in understanding the molecular genetics of thyroid cancer. Consequently, several molecular tests, that improve the accuracy of preoperative diagnosis of cytologically indeterminate thyroid nodules, have been developed and are now commercially available. Some of these tests have achieved high sensitivities and specificities among Bethesda III and IV thyroid nodules.^6^ To date, however, most of these molecular analyses are exclusively performed in the United States. Few studies reported the use of multigene sequencing assay for prospective evaluation of FNA samples in East Asia, where the genomic characterization and practices for the management of thyroid cancer may be different from western countries.^7^, ^8^ In the current study, we analyzed a multigene panel of thyroid cancer‐related genetic markers for cancer diagnosis in indeterminate thyroid nodules, then correlated the results with histology when available.

## 2 MATERIALS AND METHODS

### 2.1 Study cohorts

The prospective study was approved by the Peking University Cancer Hospital Institutional Review Board (No.2018KT101). Summarily, 1055 consecutive patients with 1193 thyroid nodules were enrolled in the study, from February 2018 to September 2019. The patients were 18 years or older, had one or more thyroid nodules with clinical and diagnostic ultrasound features, and consented to provide samples for molecular analysis.

### 2.2 Sample collection

Fine‐needle aspirations were performed by the treating surgeons under the US guidance. Briefly, a standard protocol was applied for FNAs, using a 23‐ga needle applying the non‐aspiration or aspiration technique. Specifically, samples were placed on glass slides, immediately fixed with 95% alcohol and then, stained. An additional pass of samples were also collected in a preservative, then stored at 4°C to await routine diagnostics. Categorization and diagnosis of all samples were performed according to the Bethesda System for Reporting Thyroid Cytopathology (TBSRTC). Spare samples from nodules diagnosed as Bethesda III and IV, were transported to the laboratory for molecular analysis.

The pathologists were not aware of molecular analysis results and none of the technicians involved in performing molecular analysis were aware of cytology and histopathology results. The surgeons know both of the cytology and molecular test results before treatment decision making. Indications for surgery included: (a) molecular test‐positive nodules over 1 cm in diameter or ≤1 cm willing to accept surgery; (b) molecular test‐negative nodules with compressive symptoms; (c) molecular test‐negative nodules accompanied by malignancy; and (d) highly suspicious of malignancy in image and willing to accept diagnostic surgery regardless the molecular results.

### 2.3 Molecular analysis

DNA and total RNA were isolated from FNA samples using the AllPrep DNA/RNA Mini Kit (QIAGEN), according to the manufacturers’ instructions, and their concentrations determined using a Qubit 3.0 Fluorometer（Thermo Fisher Scientific). NGS libraries were prepared from 10 ng of DNA and 10 ng of RNA, using the FSZ‐Thyroid NGS Panel V1, a NGS tool for the detection of more 1000 hotspots of 16 thyroid cancer‐related genes (AKT1, BRAF, CTNNB1, EIF1AX, EZH1, GNAS, HRAS, KRAS, NRAS, PIK3CA, RET, SPOP, TERT, TP53, TSHR, and ZNF148) and 46 types of gene fusions occurring in thyroid cancer comprising RET, PPARG, NTRK1, NTRK3, BRAF, ALK, THADA, and others in RNA. More details about the panel were described in File S1. The libraries were normalized for template preparation, on the Ion Chef, then sequenced on the Ion Proton (Thermo Fisher Scientific) platform according to the manufacturer's protocol. Data analysis and interpretation were performed using the Torrent Suite (version 5.2.2; Thermo Fisher Scientific).

Based on previous results,^9^, ^10^, ^11^, ^12^, ^13^ the association of malignancy risk stratification of thyroid nodules with different molecular alterations were categorized into three levels: High‐Risk, Low‐Risk, and Benign‐like (Table 1). A test was considered as positive if a genetic alteration was annotated with High‐Risk or Low‐Risk, and negative if Benign‐Like.

**TABLE 1:** Molecular alterations and risk of malignancy

Risk categories,%	Molecular alterations	Frequency	Malignancy rate, %	Histological diagnosis
High‐Risk N = 28 100 (20/20)	BRAF V600E	15	100 (12/12)	12 cPTC
	PIK3CA	1	– (0/0)	NA
	RET fusion	3	100 (2/2)	2cPTC^a^
	NTRK1 fusion	1	100 (1/1)	1fvPTC
	NTRK3 fusion	2	100(2/2)	1cPTC, 1fvPTC
	ALK fusion	1	100 (1/1)	1fvPTC
	KRAS (VAF ≥ 30%)	2	100 (2/2)	1cPTC, 1fvPTC
	HRAS (VAF ≥ 30%)	2	– (0/0)	NA
	NRAS (VAF ≥ 30%)	1	– (0/0)	NA
Low‐Risk N = 24 28.6 (4/14)	EIF1AX	4	0 (0/2)	2H/AN
	TSHR	2	0 (0/2)	1H/AN, 1HT
	KRAS (VAF < 30%)	4	100 (2/2)	1FTC, 1fvPTC
	HRAS (VAF < 30%)	5	0 (0/2)	2H/AN
	NRAS (VAF < 30%)	6	50% (2/4)	1cPTC, 1fvPTC, 2H/AN
	PPARG fusion	1	0 (0/1)	1H/AN
	NRAS (VAF < 30%) & EIF1AX	1	0 (0/1)	1FA
	HRAS(VAF < 30%) & EIF1AX	1	– (0/0)	NA
Benign‐like N = 136 18.0 (9/50)	EZH1	3	0 (0/1)	1 H/AN
	SPOP	8	0 (0/2)	2H/AN
	SPOP & BRAF L597Q	1	0 (0/1)	1H/AN
	ZNF148 & TERT	1	0 (0/1)	1H/AN
	No mutation	123	20 (9/45)^b^	3 FTC, 5cPTC, 1 fvPTC, 2 NIFTP, 2 FT‐UMP, 9 FA, 16 H/AN, 3HT, 4 HCA

Abbreviations:

cPTC, conventional variant of papillary thyroid carcinoma; FA, follicular adenoma; FTC, follicular thyroid carcinoma; FT‐UMP, follicular tumors of uncertain malignant potential; fvPTC, follicular variant of papillary thyroid carcinoma; H/AN, hyperplastic/adenomatous nodule; HCA, Hürthle cell adenomas; HT, Hashimoto thyroiditis; NA, not applicable; VAF: variant allele frequency.

^a^ One patient with squamous metaplasia.

^b^ If intermediate nodules as benign, the risk of malignancy is 20% (9/45), if intermediate nodules as malignant, the risk is 29% (13/45).

### 2.4 Statistical analyses

Descriptive statistics, for categorical data, were described using frequencies and percentages. Test sensitivities, specificities, positive predictive values (PPV), and negative predictive values (NPV) were then calculated at 95% confidence interval. Continuous data were presented as means, with standard deviation of the mean, or medians with interquartile range. In addition, cytological categories were correlated with mutational results, using the Fischer's exact and chi‐squared tests. Data were analyzed using SPSS software, version 22, with all values followed by *P* < .05 considered statistically significant.

## 3 RESULTS

Among the 1193 thyroid nodules, a total of 196 nodules, from the 189 patients, were cytologically indeterminate. A total of 152 (80.4%) patients were female, and the median age for the cohort was 47.8 ± 13.2 years (range 22‐78 years), whereas the median size of the eligible biopsied nodules was 1.8 ± 1.3 cm (range, 0.4‐8.6 cm).

**FIGURE 1:**
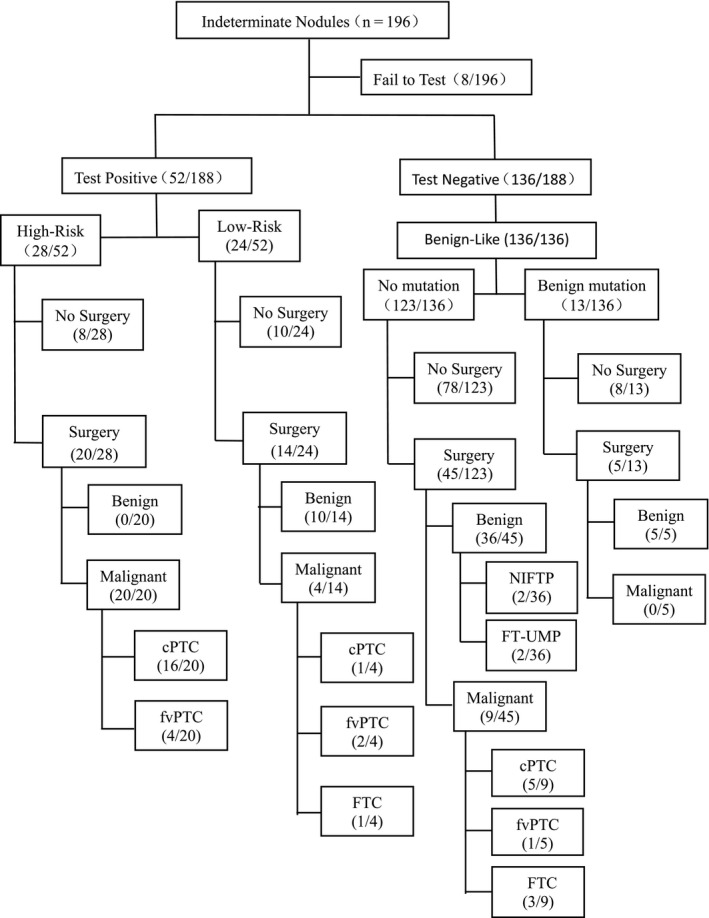
Management of indeterminate thyroid nodules. cPTC, conventional variant of papillary thyroid carcinoma; FTC, follicular thyroid carcinoma; FT‐UMP, follicular tumors of uncertain malignant potential; fvPTC, follicular variant of papillary thyroid carcinoma; NIFTP, noninvasive follicular thyroid neoplasms with papillary‐like nuclear features

Fine‐needle aspiration cytology diagnosis revealed Bethesda III and IV in 153 and 43 nodules, respectively. Out of 196 nodules, only eight failed at the pre‐sequencing step, owing to low quantity of total nucleic acid. In 52 cases, molecular alterations were identified that had High‐Risk of malignancy (n = 28) or Low‐Risk of malignancy (n = 24) and were, therefore, classified test‐positive. Test‐negative were observed in 72.3% (136/188) of these thyroid nodules, including 13 with benign mutations and 123 without any mutation. Finally, 84 nodules were resected and tested successfully. Pathological review of 84 operated nodules classified 46 (54.8%) as benign, 33 (39.3%) as malignant, and four (4.8%) as intermediate (two Noninvasive follicular thyroid neoplasms with papillary‐like nuclear features, NIFTP, and two Follicular tumors of uncertain malignant potential, FT‐UMP). Prevalence of surgery‐requiring conditions, including cancer and intermediate tumors, was 44% in the entire cohort. Histopathological analysis indicated that a majority of malignancies were papillary thyroid carcinomas (29/33; 87.9%), followed by follicular thyroid carcinoma (FTC, 4/33; 12.1%). Nonmalignant nodules included adenomas, nodular goiter, Hürthle cell adenomas, and other benign diagnoses (Figure 1).

The most common gene aberrations observed were RAS mutations (n = 22), including HRAS (n = 8), NRAS (n = 8), and KRAS (n = 6). These were followed by BRAF V600E (n = 15), whereas other positive mutations included EIF1AX, TSHR, PIK3CA, and others. We also found rearrangements in RET/CCDC6 (n = 3), ETV6/NTRK3 (n = 2), TPR/NTRK1(n = 1), PAX8/PPARG (n = 1), and STRN/ALK (n = 1). Benign mutations were detected in 6.9% (13/188) of these cases, including SPOP (n = 9), EZH1 (n = 3), and ZNF148 (n = 1) (Table 1).

The prevalence and postoperative pathology for each genetic alterations identified are summarized, and the risk of malignancy for each genetic alteration is outlined in Table 1. Based on the risk stratification criteria of molecular alterations for thyroid nodules (Table 1), 14.9% (28/188) of the nodules were classified into High‐Risk group (Bethesda III, n = 26; Bethesda IV, n = 2), 20 out of these thyroid nodules were finally surgically resected, and all of the resected nodules were malignant according to the pathological results. In the Low‐Risk category, 24 thyroid nodules were involved, 14 nodules in this group were surgically removed, and 28.6% (4/14) were malignant. A total of 136 nodules were classified Benign‐like, including 123 without any meaningful gene variation and 13 with benign mutations including SPOP, EZH1, and ZNF. Forty‐five of the 123 no mutation thyroid nodules were resected, based on the postoperative pathologic assessment, 20% (9/45) was malignant if intermediate nodules as benign, and the histopathology of these benign thyroid nodules without any mutations included 16 nodular goiter, nine follicular adenomas, four Hürthle cell adenomas, three thyroiditis, two NIFTP, and two FT‐UMP. Five of the 13 nodules in Benign‐Like category with benign mutations were resected, the pathological results were all benign. Totally, the malignancy rate in benign‐like category is 18% (9/50), if intermediate nodules counted as benign.

According to histological diagnosis, we calculated a sensitivity and specificity of 73% (95% CI 54%‐86%) and 80% (95% CI 66%‐90%), respectively. The PPV and NPV were 71% (24/34: 95% CI: 52%‐84%) and 82% (41/50; 95% CI 68%‐91%) respectively, when intermediate nodules was categorized as benign. When intermediate nodules were categorized as malignant, a sensitivity, specificity, PPV and NPV was 65% (95% CI 47%‐79%), 79% (95% CI 64%‐89%), 71% (24/34:95% CI: 52%‐84%), and 74% (37/50; 95% CI, 59%‐85%), respectively, were obtained (Table 2).

**TABLE 2:** The performance of multigene testing

1. All ITN (Bethesda Ⅲ+Ⅳ, n = 84)						
A. Intermediate nodules as benign						
		Histological diagnosis				Sensitivity: 73% (54‐86) Specificity: 80% (66‐90) PPV: 71% (52‐84) NPV: 82% (68‐91)
		Malignant		Benign		
Molecular test	Positive	24		10		
	Negative	9		41		
Prevalence of malignancy: 39%						
B. Intermediate nodules as malignant						
		Histological diagnosis			Sensitivity: 65% (47‐79) Specificity: 79% (64‐89) PPV: 71% (52‐84) NPV: 74% (59‐85)	
		Malignant	Benign			
Molecular test	Positive	24	10			
	Negative	13	37			
Prevalence of malignancy: 44%						

2. Bethesda Ⅲ category (n = 56)				
A. Intermediate nodules as benign				
		Histological diagnosis		Sensitivity: 81% (61‐93) Specificity: 83% (64‐93) PPV: 81% (61‐93) NPV: 83% (64‐93)
		Malignant	Benign	
Molecular test	Positive	22	5	
	Negative	5	24	
Prevalence of malignancy: 48%				
B. Intermediate nodules as malignant				
		Histological diagnosis		Sensitivity: 79% (59‐91) Specificity: 82% (62‐93) PPV: 81% (61‐93) NPV: 79% (60‐91)
		Malignant	Benign	
Molecular test	Positive	22	5	
	Negative	6	23	
Prevalence of malignancy: 50%				

3. Bethesda Ⅳ category (n = 28)				
A. Intermediate nodules as benign				
		Histological diagnosis		Sensitivity: 33% (6‐76) Specificity: 77% (54‐91) PPV: 29% (5‐70) NPV: 81% (57‐94)
		Malignant	Benign	
Molecular test	Positive	2	5	
	Negative	4	17	
Prevalence of malignancy: 21%				
B. Intermediate nodules as malignant				
		Histological diagnosis		Sensitivity: 22% (4‐60) Specificity: 74% (49‐90) PPV: 29% (5‐70) NPV: 67% (43‐85)
		Malignant	Benign	
Molecular test	Positive	2	5	
	Negative	7	14	
Prevalence of malignancy: 32%				

Abbreviations: NPV, negative predictive value; PPV, positive predictive value.

Multigene testing performance was better in Bethesda Ⅲ than Ⅳ specimens when intermediate nodules counted as malignant (*P* = .037).When intermediate nodules counted as benign, the differences were not statistically significant (*P* = .171).

In total, nine malignant nodules were negative for all tested mutations in our cohort, including five in Bethesda III and four in Bethesda IV. All these patients had surgery due to suspicious imaging features. The clinicopathologic characteristics of these nodules are listed in Table 3. All of the Bethesda Ⅲ and one of the four Bethesda Ⅳ nodules were papillary thyroid microcarcinoma (PTMC) without lymph node metastasis except one, whose lateral neck lymph nodes were involved. The other three Bethesda Ⅳ nodules revealed to be FTC without vascular invasion after diagnostic surgery.

**TABLE 3:** Clinicopathologic characteristics of the nine test‐negative malignant nodules

Patient	Sex	Age	TBSRTC	Ultrasound characteristics	Size^a^	Histology	ETE	LNM
1	F	42	III	Hypoechoic, taller than wide, irregular margins	0.8	fvPTC	No	No
2	F	60	III	Hypoechoic, irregular margins, microcalcifications	0.5	fvPTC	No	No
3	M	25	III	Hypoechoic, irregular margins, microcalcifications	0.6	cPTC	No	No
4	F	42	III	Hypoechoic, taller than wide, irregular margins, microcalcifications	0.7	cPTC	No	No
5	F	56	III	Hypoechoic, microcalcifications, lymphadenopathy	0.6	cPTC	No	Yes
6	F	30	IV	Hypoechoic, irregular margins, microcalcifications	0.8	cPTC	No	No
7	F	37	IV	Hypoechoic, irregular margins, microcalcifications, intranodular vascularity	1.7	FTC	No	No
8	M	36	IV	Hypoechoic, irregular margins, intranodular vascularity	2.8	FTC	No	No
9	F	49	IV	Hypoechoic, irregular margins, eggshell calcification, intranodular vascularity	3.2	FTC	No	No

Abbreviations: ETE, extrathyroidal extension; LNM: lymph node metastesis; TBSRTC, The Bethesda system for reporting thyroid cytopatjology.

^a^ Measured in pathological specimen.

## 4 DISCUSSION

The current version “American Thyroid Association (ATA)” guidelines clearly advocates the use of molecular markers for validation of samples that turn indeterminate following cytological assay.^1^ Numerous studies have characterized indeterminate thyroid nodules by analyzing BRAF V600E mutation.^14^ However, this method has been found to generate limited sensitivity, for low incidence in BRAF mutation in Bethesda III/IV nodules. To circumvent this issue, researchers are increasingly turning to simultaneous analysis of panels of markers, rather than single‐gene mutation.^15^ There are various molecular platforms available in recent years, the Afirma GEC (Veracyte. South San Francisco,CA) based on mRNA expression pattern; the ThyroSeq test (CBL path Inc., North Haven, CT) evaluating genes associated with thyroid cancer, the ThyGenX/ThyraMIR (Interpace Diagnostics, Parsippany, NY) that combines the genetic alterations with an miRNAs classifier; and so on. The meta‐analyses conducted to evaluate these molecular tests for indeterminate thyroid lesions revealed pooled sensitivity of 84%‐98%, specificity of 12%‐92%, NPV of 91%‐96%, and PPV of 37%‐78%.^16^, ^17^ Unfortunately, these clinically validated commercial ancillary molecular tests can only be available in a few developed nations. Samples from other countries need to be transported across large distances entailing high costs.^18^ Only a handful of studies describing these methods were published outside USA. The current study aimed to use high throughput sequencing to evaluate the indeterminate FNA samples, owing to the paucity of information in China and East Asia, where overtreatment of thyroid nodules has become a social problem.^19^, ^20^

Previous studies have reported the differences between Asian and Western genetic characteristics, with regards to thyroid nodules and thyroid cancer. For example, prevalence of the BRAF V600E point mutation was found to be higher in Asian PTC populations, relative to those in Western countries, whereas that of RAS mutations are lower.^7^, ^21^, ^22^ In the present study, RAS mutations were also most commonly encountered in Bethesda III/IV nodules, followed by BRAF V600E mutation. A previous study evaluating ThyroSeq v2 across three institutions, reported lower incidence of BRAF mutation and higher RAS mutation albeit with no statistical differences (5/156 vs 15/188, *P* = .067; 29/156 vs 22/188, *P* = .093).^9^ A similar prevalence of RAS mutation (19/190, *P* = .623), was observed in another study, performed at a single institution, yet a significantly lower BRAF V600E mutation was observed (2/188, *P* = .002).^23^

BRAF V600E is the most commonly detected mutation in thyroid cancers and is specifically associated with papillary thyroid carcinoma.^24^ Moreover, follicular patterned lesions are the most difficult to differentiate, because the follicular variant of papillary thyroid carcinoma, FTC, the benign follicular thyroid adenoma, and borderline lesions can share the same RAS‐like molecular alterations.^13^, ^25^ Consequently, numerous studies have shown that RAS mutations are not specific to thyroid malignancy, owing to a wide range of PPV, such as 0%‐100%, 28%‐100%, and 0%‐100% observed in Bethesda III, IV, and V nodules, respectively.^26^ This has been the main factor affecting PPV of the panels. A previous systematic review found that 607 of 2674 nodules contained a RAS variant, that had a corresponding 66% PPV.^27^ In the current study, we found that half of the RAS‐mutation nodules were resected, revealed that 60% were malignant, consistent with previous studies. When variants were specified across RAS genes, KRAS had the highest malignant rate. There used to be hypothesis that RAS‐mutated follicular adenomas may be precursors of thyroid carcinoma.^28^, ^29^ However, a recent study revealed that RAS mutations, alone, are not effective markers for detecting malignancies among Bethesda III/IV classes, although they may predict favorable behavior.^30^ Based on these findings, further research is needed to determine the diagnostic value of RAS mutations in indeterminate thyroid nodules.

Apart from RAS mutations, EIF1AX and TSHR mutations also showed false positives following molecular analysis. EIF1AX mutations have been documented in papillary, poorly differentiated, and anaplastic thyroid carcinomas as well as in benign thyroid nodules.^31^, ^32^ Karunamurthy et al^33^ found five EIF1AX‐positive FNA samples during surgical follow‐up, one nodule was PTC, whereas the others were benign follicular adenomas or hyperplastic nodules. Moreover, low frequency of TSHR mutation has been reported, with most TSHR‐positive nodules found to be benign following surgery.^23^, ^34^ In the current study, we found four EIF1AX and two TSHR mutations, with postoperative pathology of two EIF1AX‐and two TSHR‐mutated nodules conferring no risk of cancer. These results indicate that caution should be taken when including these Low‐Risk markers in diagnostic decisions.

Previous genomic evidence indicates that PTC and benign nodules have independent origins. Part of adenomatoid nodule carried mutually exclusive SPOP, EZH1, and ZNF148 mutations, a unique molecular signature that differs from PTC.^10^ In order to improve the ability of exclusion diagnosis, we added these characteristic mutations to the panel. SPOP, EZH1, and ZNF148 mutations were found in 8, 3, and 1 nodules, respectively. Surgical resection performed on six of these nodules indicated that all of them were benign, including one nodule accompanied by TERT promoter mutation. Generally, presence of a TERT promoter mutation leads to a definite diagnose of thyroid cancer,^35^ although a rare case of TERT promoter mutation was previously reported in a benign follicular adenoma that occurred concurrently with other mutations.^36^ From our results, it is evident that these benign markers are reliable.

Our results also revealed a higher PPV and lower NPV in present cohort, relative to what has previously been reported in ThyroSeq v2 studies.^9^, ^23^ This can be attributed to: First, a higher cancer prevalence in our study relative to previous reports. Second, presence of a higher frequency of BRAF V600E mutation, with a 100% risk of malignancy. Third, positive nodules in Low‐Risk category had a low rate of malignancy as discussed above.

Specifically, nine malignant cases returned negative results following the molecular assay did not show any mutation or fusion. Six of them were PTMCs and five were Low‐Risk eligible for active surveillance.^1^ The high proportion of PTMC in false‐negative group suggested that the size of the thyroid nodules may have an impact on molecular testing result. The false negative FTCs were intrathyroidal, well differentiated without vascular invasion. Similarly, all the intermediate nodules yielded a negative test result. These indicated that neoplasms without identifiable mutations may tend to grow in a indolent pattern.

Previous studies have shown that only 26% of indeterminate nodules were positive for at least one variant and/or fusion,^27^ which is similar with our results. Therefore, evaluation of genomic variants or fusions in DNA and/or RNA could miss some malignant lesions. Even if the number of genes and sites detected are increasing, only a few variant or fusion have been reported with confirmative predictive value. The new generation testing method evaluates the alteration in copy number, as well as abnormal gene expression, rather than point mutations and gene fusions, thereby improving the efficiency in a prospective double‐blinded multicenter validation study.^37^ This indicated molecular detection beyond mutations and fusions may provide future differentiation of Low‐Risk or test‐negative nodules.

This study had several limitations. First, the relatively small surgical sample size reduced the reliability of results. Second, by selecting patients based on cytology, molecular testing, and ultrasound imaging for surgery, the prevalence of malignancy was increased. There is a reason to believe that the unresected test‐negative nodules have a lower risk of malignancy, therefore, the specificity and NPV were underestimated.

## 5 CONCLUSIONS

In conclusion, the present study highlights the potential impact of NGS‐based screening for effective diagnosis of patients with thyroid indeterminate FNA. The resulting data are expected to allow for further stratification of patients, according to their risk of malignancy, and refine corresponding treatment options. Furthermore, benign mutations such as SPOP, EZH1, and ZNF148 may be used as exclusive diagnostic markers.

## CONFLICT OF INTEREST

The authors declare that they have no competing interests.

## AUTHOR CONTRIBUTIONS

**Yuntao Song**: designed the study, performed fine‐needle aspiration and surgical procedures, performed the statistical analysis, interpreted the data; **Guohui Xu, Hao Yu and Tianxiao Wang**: performed fine‐needle aspiration and surgical procedures; **Tonghui Ma**: designed the Next‐generation sequencing procedure; **Yanli Zhu**: performed the cytopathology examination; **Wenbin Yu, Wei Wei**: performed surgical procedures; **Bin Zhang**: designed the study, performed surgical procedures, and revised the manuscript. The manuscript was written by Yuntao Song with input from all of the authors.

## Supporting information

Supplementary Material

## Data Availability

The authors confirm that the article contains a Data Availability Statement even if no data are available (list of sample statements) unless the article type does not require one. The authors confirm that they have included a citation for available data in the references section, unless the article type is exempt.
