# Supplementary material for: Utility of a multigene testing for preoperative evaluation of indeterminate thyroid nodules: A prospective blinded single center study in China
Source: Cancer Med. 2020 Sep 25;9(22):8397–405. doi: 10.1002/cam4.3450 (PMC7666727; doi:10.1002/cam4.3450)
Supplement: Supplementary file 1 — Supplementary Material [file CAM4-9-8397-s001.docx]

Details about FSZ-Thyroid NGS Panel V1

Multiplex PCR Targeted Amplicon Sequencing

The genes in FSZ-Thyroid NGS Panel V1 was selected according to the previous research results ^1-6^ and genomic data of TCGA ^7^(The Cancer Genome Atlas). The panel was designed to maximize the number of unique driver genes of thyroid cancer by limited number of multiplex PCR targeted amplicons. Primers were selected for detecting genomic alterations in thyroid cancers as well as barcoding adapter DNA oligos by a customized procedure to balance coverage, Tm, dimmer potential, and predicted specificity with the human genome.

Sequencing libraries were generated using one-step multiplex PCR targeted amplicons, Briefly, DNA and RNA were isolated from thyroid nodules FNA samples with the AllPrep DNA/RNA mini Kit(QIAGEN), The concentration of DNA and RNA were measured by Qubit 3.0(Thermo Fisher Scientific).10ng RNA were transcript to cDNA by SuperScript III Reverse Transcriptase (Thermo Fisher Scientific). Two 20μl reaction pools, which containing 10μl KAPA2G Fast PCR (Roche), 2.0ul primers mixtures, 10ng sample DNA template (or cDNA template above ) and distilled water up to desired volume , were mixed to detect driver gene variants in DNA or gene fusions in cDNA. Initial denaturation was at 98℃ for 30s, followed by 22 cycles at 98℃ for 10s, 60℃ for 90s, and 72℃ for 90s, and a final elongation at 72℃ for 10min. Each reaction pools were cleaned using Agencourt AMPure XP 60mL kit (Beckman A63881) to remove unused primers, according to the manufacturer’s specifications. The concentration of the barcoded PCR produced library was then measured by Qubit 3.0(Thermo Fisher Scientific), and diluted to 100pMol. 20ul pooled amplicons were sequenced on the Ion Proton system (Thermo Fisher Scientific).

Local alignments of reads to the hg19 genome were performed using bowtie2 (version 2.2.4) in paired-end mode. SAM alignment files were converted to BAM files, sorted and indexed using samtools (version 0.1.19). BAM files were processed with bam-readcount Bam-readcount outputs were processed with a custom written Perl script.

Primers for genes mutation in DNA :

16 genes were included: BRAF, KRAS, NRAS, HRAS, TERT, EIF1AX, TP53, CTNNB1, RET, EZH1, SPOP, ZNF148, GNAS, TSHR, AKT1,PI3KCA

| Gene | Forward Primer | Reverse Primer |
| --- | --- | --- |
| HRAS | CCTGTACTGGTGGATGTCCTCA | GCCTGTTGGACATCCTGGATAC |
| HRAS | CGCCAGGCTCACCTCTATAG | AGGAGCGATGACGGAATATAAGC |
| EIF1AX | ACGTCATCAATATCTTCATCATCATCTCC | AAATTACAGTGCTGACTTATGAGTATTTCTTAC |
| E1F1AX | ACTTACCCTGACCATCCTCTTTGAA | ACAGATAATTAATGTCATTTACCTCCTTTTCT |
| EIF1AX | GACCTGCAATCTACGGGCA | CCGCTACCCGGAAAGAAGTC |
| KRAS | CATGTACTGGTCCCTCATTGCA | GTAATAATCCAGACTGTGTTTCTCCCTT |
| KRAS | TACCTCTATTGTTGGATCATATTCGTCCA | TATTATAAGGCCTGCTGAAAATGACTGAAT |
| CTNNB1 | GTCACTGGCAGCAACAGTCTTA | CCCTGTTCCCACTCATACAGGA |
| EZH1 | TAATTCAAAGAAGCCTGGCCCACAGAA | GGAGTCTGTGCAGAAGAACGAATT |
| EZH1 | CACAGGTCAGGGTCACATTCTC | TAGCTTATTTCCCTTCTAAGACTTACCTGT |
| RET | ATTAAAGCTGGCTATGGCACCT | GATGTGCTGTTGAGACCTCTGT |
| RET | GTGCCAAGCCTCACACCAC | ACTTGTGGTAGCAGTGGATGC |
| RET | CCATCCTGACCTGGTATGGTCA | CCTGCTTCAGGACGTTGAACTC |
| RET | GGTCAACCACCCACATGTCA | AAGAGGGAGAACAGGGCTGTA |
| RET | GCTTCAAGGTCTGCGCTCT | CAGCTTGGGTCTTCCAGGAG |
| RET | AGGCCCGCTCCTCCT | GTGAGGGCCCGCTCATC |
| RET | CAGCTCGTTCATCGGGACTT | ACCTGGCTCCTCTTCACGTA |
| RET | CCTCCTTCCTAGAGAGTTAGAGTAACT | CACCCACACTTACACATCACTTTG |
| SPOP | CCTTGGCATTCAGGATGGAGAAT | TTTGGACAGGTGTTTGCGAGTA |
| GNAS | TGCTTTAGATTGGCAATTATTACTGTTTCG | GTTGACTTTGTCCACCTGGAACT |
| GNAS | TCCCTCTGGAATAACCAGCTGT | AAGCAAAGCGTTCTTTACGAACAG |
| TSHR | CTCTCCTGGGCAATGTCTTTGT | GCGATGAGGAGCAGGTACATC |
| TSHR | CTCATCGCCTCTGTAGACCTCT | GCGTATACACCGATAACTCGCTT |
| TSHR | CCTTTGGTGGGAATAAGTAGCTATGC | ACATAACAGCAGCAGACGATGA |
| TSHR | TATGTGAAGATCTACATCACAGTCCGAA | GCTGACAGAGCATAGAATGAGATTGG |
| AKT1 | CCGCTCCTTGTAGCCAATGA | GGGTCTGACGGGTAGAGTGT |
| NRAS | GAGGTTAATATCCGCAAATGACTTGC | AAACCTGTTTGTTGGACATACTGGA |
| NRAS | GTGGGATCATATTCATCTACAAAGTGGT | GATTACTGGTTTCCAACAGGTTCTTG |
| BRAF | TCAGTGGAAAAATAGCCTCAATTCTTACC | CTTCATGAAGACCTCACAGTAAAAATAGGT |
| PIK3CA | CCCTCCATCAACTTCTTCAAGATGA | ATTACTTTTAAAAAGGGTTGAAAAAGCCGA |
| PIK3CA | AATTGAACCAGTAGGCAACCGT | TTGGTTTAGACAGAAATATTTTAGAAAGGGACA |
| PIK3CA | CTTCCAAATCTACAGAGTTCCCTGTT | TAACCATATCAAATTCACACACTGGCAT |
| PIK3CA | TACAGAGTAACAGACTAGCTAGAGACAATG | TCTCCATTTTAGCACTTACCTGTGAC |
| PIK3CA | TTTGATGACATTGCATACATTCGAAAGAC | ATTGTGTGGAAGATCCAATCCATTTTTG |
| TERT | CACCCGTCCTGCCCCTTCAC | GCGGAAAGGAAGGGGAGG |
| ZNF148 | CTGTGGCAAGTGTCATTGATGAA | GATATCTCATGCTGTCCATTAGCTTT |
| TP53 | CTTGAACCATCTTTTAACTCAGGTACTGT | GCCTGGGCATCCTTGAGTTC |
| TP53 | CTACTGGGACGGAACAGCTT | CTTGCTTACCTCGCTTAGTGCT |
| TP53 | TCTTGGGCCTGTGTTATCTCCTA | CCTGACCTGGAGTCTTCCAGT |
| TP53 | GGCCTCTGATTCCTCACTGATTG | TCATAGGGCACCACCACACTAT |
| TP53 | AGCTGTGGGTTGATTCCACA | CAGCTGCTCACCATCGCTAT |
| TP53 | GAAGCTCCCAGAATGCCAGA | GTAGCTGCCCTGGTAGGTTT |
| TP53 | GTTGGAAGTGTCTCATGCTGGAT | CCATAGGTCTGAAAATGTTTCCTGACT |
| TP53 | GCATTCTGGGACAGCCAAG | TACGGCCAGGCATTGAAGT |

Primers for gene fusions in RNA :

| Gene | Primers： |
| --- | --- |
| THADA | GCACGAGCTGACTGACATCA |
| THADA | AAACCGTGACAACTGCCATG |
| THADA | CCAGCTGCACTGACCAGT |
| THADA | GATGGATGGTACTTCTTCTGCTCTC |
| THADA | TCCATGTTTGGTGACCAGAGC |
| IGF2BP | CCACTGTAAATGAGGCGGGATAT |
| IGF2BP | TTCTATGGGTTTCCCGTGCAG |
| LOC389473 | CCTGAGTTTCTTGTGCATTCCTC |
| NTRK1 | CTGCCTTCATGGACAACCCT |
| NTRK1 | TCCTTCTTCTCCACCGGGT |
| NTRK3 | CCCTCACCCAGTTCTCGC |
| NTRK3 | GTGGTTGATGTGGTGCAGTG |
| ETV6 | AACCACATCATGGTCTCTGTCT |
| ETV6 | GAAACTCTATACACACACAGCCGG |
| TPM3 | CGTGCTGAGTTTGCTGAGAGAT |
| TPM3 | TCCAGCATCCTTTGTGTACAGAG |
| SQSTM1 | GAACGTTGGGGAGAGTGTGG |
| TPR | ACATTGAAACAGCACCTCAGTAATATG |
| IRF2BP2 | TGACTGCAGGCAGGTTGTT |
| BRAF | TCCTCATCAGCTCCCAATGTG |
| BRAF | CCCACTGTAATCTGCCCATCAG |
| BRAF | GGGCTGTGGAATTGGAATGGA |
| BRAF | GATCCTCCATCACCACGAAATCC |
| OSBPL9 | TCATGCATCTCTTGTCGCAGG |
| SND1 | GGCTTGGTGCAGGAAGGA |
| NFYA | GAGCTAAACTAGAGGCAGAAGGGA |
| PICALM | ACGCAACCAACCTTAATATACAGC |
| AKAP9 | AGAGCAACTCAACCAAGTGAAAATG |
| RBMS3 | CTGACACAGCAGATGAATCACC |
| AGK | CTCTGCCTGCTGACCTGG |
| TANK | GACTCTTGATCAGCCACAAGATAAAG |
| LMO7 | CAAAGCAACCCGTACTATAATGGTC |
| KLHL7 | TGTCAAGCGAGTAACACATCTTCTC |
| KIAA1549 | CGGCTCATCACCACAGACA |
| KIAA1549 | GCACAGATGCAGATCGACAAG |
| RET | CTGCCCAGTACCTACTCCCT |
| RET | GGACAAAAGGCCGGTGTTTG |
| RET | TCCAAGAACCAAGTTCTTCCGAG |
| RET | GGTGCCATAGCCAGCTTT |
| RET | ACCGAGACGATGAAGGAGAAGA |
| RET | CACCAGAGGAATGCAGCTTGT |
| RET | CTGAGAGGCCGTCGTCATAAAT |
| PRKAR1A | TGTGAAATTGTGGGGCATCGA |
| CCDC6 | CACTGCAGGAGGAGAACCG |
| TRIM33 | TAGTAAGAAGGGGAAAACTGCGC |
| AFAP1L2 | GCCAAGCAGCTCTGTGTCA |
| KIAA1217 | GCTTTCCTCCTGCGTCAAG |
| CCDC186 | AACACAAGCAAAGGAAGAAGCAG |
| NCOA4 | GTTATCAAGCTCCTTACATACCCAGC |
| ALK | CTCAGCTTGTACTCAGGGCTC |
| GFPT1 | GTGATCCCTTTACAGTTGCTGG |
| STRN | CCACAAGTTGAAATACGGGACAG |
| PPARG | AGTGGTCTTCCATTACGGAGAG |
| PAX8 | CAGCTATGCCTCCTCTGCC |
| PAX8 | CAGTTCACGGGCCAGGC |
| PAX8 | CAACCTCTCGACTCACCAGAC |
| PAX8 | CCTATGCCTCCCCCAGC |
| CREB3L3 | CCTTCACCCACATTACCACCA |

|  | (20 ul total ) | Condition |
| --- | --- | --- |
| ddH2O | up to desired volume | 98℃ 30s  22 cycles:  {98℃ 10s  60℃ 90s  72℃ 90s}  72℃ 10min |
| KAPA2G Fast PCR Mix | 10 ul |  |
| Primer | 2.0 ul |  |
| template | 20ng |  |
| Total | 20 ul |  |

Reaction Condition of the test

REFERENCES:

1. Bychkov A. Prevalence of BRAFV600E mutation in Asian patients with thyroid cancer. (Letter to editor). *Malaysian J Pathol.* 2017; 39: 95–6.
2. Liang J , Cai W , Feng D , et al. Genetic landscape of papillary thyroid carcinoma in the Chinese population. *Journal of Pathology*, 2018; 244: 215–226
3. [Valderrabano P](http://pubmed.cn/javascript:void(0);) , [Khazai L](http://pubmed.cn/javascript:void(0);) , [Leon ME](http://pubmed.cn/javascript:void(0);) , et al. Evaluation of ThyroSeq v2 performance in thyroid nodules with indeterminate cytology. *[Endocr Relat Cancer](http://pubmed.cn/search?q='Endocrine-related%20cancer'%5bjournal%5d)* 2017 03;24 (3): 127-136
4. Eszlinger M, Krogdahl A, Munz S et al. Impact of molecular screening for point mutations and rearrangements in routine air-dried fine needle aspiration samples of thyroid nodules. *Thyroid* 2014; 24; 305–313
5. Zhu Z, Gandhi M, Nikiforova MN, Fischer AH, Nikiforov YE. Molecular profile and clinical pathologic features of the follicular variant of papillary thyroid carcinoma. An unusually high prevalence of ras mutations. *Am. J. Clin. Pathol.* 2003; 120; 71–77.
6. Ye L , Zhou X , Huang F , et al. The genetic landscape of benign thyroid nodules revealed by whole exome and transcriptome sequencing. *Nature Communications,* 2017, 8:15533.
7. [Cancer Genome Atlas Research Network](https://pubmed.ncbi.nlm.nih.gov/?term=Cancer+Genome+Atlas+Research+Network%5bCorporate+Author%5d). Integrated Genomic Characterization of Papillary Thyroid Carcinoma. *Cell*. 2014. 159. 676-90.
